# The making of multivalent gamma delta TCR anti-CD3 bispecific T cell engagers

**DOI:** 10.3389/fimmu.2022.1052090

**Published:** 2023-01-05

**Authors:** Eline van Diest, Mara J. T. Nicolasen, Lovro Kramer, Jiali Zheng, Patricia Hernández-López, Dennis X. Beringer, Jürgen Kuball

**Affiliations:** ^1^ Center for Translational Immunology, University Medical Center Utrecht, Utrecht University, Utrecht, Netherlands; ^2^ Department of Hematology, University Medical Center Utrecht, Utrecht University, Utrecht, Netherlands

**Keywords:** tumor immunology, bispecific T cell engager, gamma delta TCR, protein engineering, Gamma Delta T cells

## Abstract

**Introduction:**

We have recently developed a novel T cell engager concept by utilizing γ9δ2TCR as tumor targeting domain, named gamma delta TCR anti-CD3 bispecific molecule (GAB), targeting the phosphoantigen-dependent orchestration of BTN2A1 and BTN3A1 at the surface of cancer cells. GABs are made by the fusion of the ectodomains of a γδTCR to an anti-CD3 single chain variable fragment (scFv) (γδECTO-αCD3), here we explore alternative designs with the aim to enhance GAB effectivity.

**Methods:**

The first alternative design was made by linking the variable domains of the γ and δ chain to an anti-CD3 scFv (γδVAR-αCD3). The second alternative design was multimerizing γδVAR-αCD3 proteins to increase the tumor binding valency. Both designs were expressed and purified and the potency to target tumor cells by T cells of the alternative designs was compared to γδECTO-αCD3, in T cell activation and cytotoxicity assays.

**Results and discussion:**

The γδVAR-αCD3 proteins were poorly expressed, and while the addition of stabilizing mutations based on finding for αβ single chain formats increased expression, generation of meaningful amounts of γδVAR-αCD3 protein was not possible. As an alternative strategy, we explored the natural properties of the original GAB design (γδECTO-αCD3), and observed the spontaneous formation of γδECTO-αCD3-monomers and -dimers during expression. We successfully enhanced the fraction of γδECTO-αCD3-dimers by shortening the linker length between the heavy and light chain in the anti-CD3 scFv, though this also decreased protein yield by 50%. Finally, we formally demonstrated with purified γδECTO-αCD3-dimers and -monomers, that γδECTO-αCD3-dimers are superior in function when compared to similar concentrations of monomers, and do not induce T cell activation without simultaneous tumor engagement. In conclusion, a γδECTO-αCD3-dimer based GAB design has great potential, though protein production needs to be further optimized before preclinical and clinical testing.

## Introduction

During the last decade, the introduction of immunotherapy has led to a significant improvement in treatment options for cancer patients. Many of these therapies aim to improve T lymphocyte mediated tumor recognition, for example by relieving the breaks on these cells by checkpoint inhibition, or by arming T cells with chimeric antigen receptors that induce cancer cell recognition (1). Another opportunity to use T cells for cancer therapy arose from the discovery that T cells can be redirected to tumor cells by a bispecific hybrid antibody (2), and since this initial discovery, many different bispecific antibodies to redirect T cells towards tumor cells have been developed (3). In general, bispecific antibodies combine a tumor binding domain, directed to a tumor associated antigen, with a T cell recruitment domain, most often binding to CD3ϵ. These bispecific antibodies, also called T cell engagers (TCE), can induce T cell mediated cytotoxicity towards tumor cells by simultaneously binding to the target antigen and CD3, without specific T cell receptor (TCR) - MHC engagement (4). Blinatumomab, a TCE directed against CD19 and CD3 is the first TCE construct that is FDA approved for the treatment of patients with refractory or relapsed pre-B-acute lymphoid leukemia (5). Recently a second TCE, Tebentafusp, targeting a gp100 peptide in HLA-A*02:01 and CD3, was FDA approved for the treatment of unresectable or metastatic uveal melanoma (6). Next to these two TCEs, a plethora of novel TCEs with different designs and tumor targets is currently in various stages of clinical development (7, 8).

The majority of TCEs utilize antibody-derived tumor binding domains, in the form of single chain variable fragments, antigen binding fragments, or full length antibodies (9). These antibody-derived binding domains can be engineered to bind to tumor associated antigens with very high affinity, which has been reported as beneficial for the development of highly potent TCEs (10, 11). A challenge that remains, however, is the selection of novel suitable target antigens for TCEs. On-target off- tumor toxicity remains a concern for high affinity TCEs when low levels of the target antigen are expressed on healthy tissue (12).

Most recently, we have developed a novel TCE concept, so called **g**amma delta TCR **a**nti-CD3 **b**ispecific molecules (**GABs**) by fusing ectodomains of a γδ T cell receptor (TCR) to an anti-CD3 single chain variable fragment (γδ_ECTO_-αCD3) (13). This concept is based on the anti-tumor activity of γ9δ2 T cells, which are important players in the recognition of foreign pathogens, virally infected cells, and also cancer cells (14). Vγ9δ2 T cells, a specific γδT cell subset mainly found in the blood, recognize members of the butyrophilin (BTN) family, namely BTN2A1, through the gamma chain of their Vγ9δ2TCR, and additionally require BTN3A1 expression on the tumor cells for full activation (15–17). Recognition of the BTN2A1-BTN3A1 complex is induced by an intra-cellular accumulation of phosphoantigens (pAg) that can bind to the intracellular B30.2 domain of BTN3A1, which is modulated by RhoB (18, 19). pAg accumulation can be caused by microbial infection, but is also associated with cancerous transformation of cells (20). *In vitro* Vγ9δ2T cells recognize and lyse a broad spectrum of solid and hematological tumor cells (21, 22) and therefore provide an interesting tool box for the development of anti-cancer therapies (23). However, the activity of Vγ9δ2T cells is diverse when analyzed in a clonal population (17), and can be hampered by many inhibitory receptors, like NKG2A (24).

GABs are a means to utilize the favorable clonal properties of natural Vγ9δ2T cells, and, by engaging mainly αβ T lymphocytes, make it possible to overcome the general poor functionally of Vγ9δ2T cells in advanced stage cancer patients. Furthermore, GAB mediated tumor recognition is independent of the mutational load or tumor associated antigen expression of the tumor cells, thus introduces a novel tumor targeting concept to the TCE field. This concept would also overcome extensive and expensive T cell engineering concepts with defined Vγ9δ2TCRs (23, 25).

Critical for the GAB concept remains the rather low affinity of the Vγ9δ2 TCR for its ligands, which has been reported in the µM range (15, 16), a couple of magnitudes lower than the high affinity antibody derived domains generally used for tumor binding in TCEs. For αβTCR based TCEs, like Tebentafusp, the consensus is that affinity maturation of the αβTCR from µM to pM is required to create a functional TCE (26). While we have shown that for the GAB, affinity maturation of the γδTCR is not essential when naturally selected high affinity CDR3 sequences of the δ chain are used (13), we hypothesized that increasing the tumor binding avidity of the Vγ9δ2 TCR would further improve the effectivity of a GAB.

Most TCEs combine only one tumor- and one T cell engaging domain, similar to our original GAB design, however there are also higher valency constructs currently being developed (9, 27, 28). Often the rationale behind the use of these higher valency constructs is to increase the potency of the TCE by increasing the tumor binding avidity rather than the direct affinity maturation of the tumor binding domain (29). In this light,

we report here on the failures and success of different strategies to create multivalent GABs, and show that while attempts to express the γ and δ variable domains as a single chain linked to an anti-CD3 single chain variable fragment (γδ_VAR_αCD3) were not successful, we observed γδ_ECTO_-αCD3-dimers as a side product during the production process with the original γδ_ECTO_-αCD3 GAB design, incorporating the full length γδTCR ectodomains. Although it is a technical challenge to achieve meaningful yields of γδ_ECTO_-αCD3-dimers, γδ_ECTO_-αCD3-dimers have improved *in vitro* potency compared to the monomeric form, while there is no evidence for non-specific T cell activation by bivalent CD3 engagement.

## Material and methods

### Generation of bispecific constructs

Design of the original γδ_ECTO_-αCD3 construct was reported previously (13). To force dimerization, the 3(G4S) linker between the OKT3 variable heavy and light chain was replaced by a G4S linker. To create the γδ_VAR_-αCD3, the variable domains of the γ and δ chain linked to an anti-CD3 single chain variable fragment were cloned into a modified pcDNA3 vector (kind gift from protein facility LTI; UMCU) using BswI and SalI restriction sites, containing a 3’ biotin acceptor peptide and His-tag after the SalI restriction site. From the N- to C-terminus the γδ_VAR_ –αCD3 had the following design, Vδ-3(G4S)-Vγ-3(G4S)-VH-3(G4S)-VL. For constructing the single chain γδ_VAR_ the C-terminus of the Vδ chain was linked to the N-terminus Vγ chain by a flexible linker with the sequence GSADDAKKDAAKKDGKS. Unless indicated otherwise, the TCR sequences used for the GAB constructs are derived from CL5 TCR (30) (γδ_VAR_ and γδ_VAR_-αCD3) or AJ8 TCR (γδ_ECTO_-αCD3) (13). CDR3 sequences of all the TCRs used are indicated in **Table 1**. The anti CD3 single chain variable fragment (αCD3) was derived from the mAb OKT3 (32).

**Table 1:** GAB sequences. Depicted are sequences used for generation of yδ_ecto_-αCD3.

GAB	REF	CDR3δ	CDR3γ
AJ8	(13)	CACDTAGGSWDTRQMFF	CALWEAQQELGKKIKVF
LM1	(30)	CACDTLLATDKLIF	CALWEAQQELGKKIKVF
A3	(17)	CACDAWGHTDKLIF	CALWEAQQELGKKIKVF
C4	(17)	CACDTLALGDTDKLIF	CALWEAQQELGKKIKVF
C5	(17)	CACDLLAPGDTSFTDKLIF	CALWEAQQELGKKIKVF
C7	(17)	CACDMGDASSWDTRQMFF	CALWEAQQELGKKIKVF
A3	(17)	CACDAWGHTDKLIF	CALWEAQQELGKKIKVF
CL5	(30)	CACDALKRTDTDKLIF	CALWEIQELGKKIKVF
6_2	(13)	CACDTLPGAGGADKLIF	CALWEVQELGKKIKVF
EPCR reactive y4δ5 TCR	(31)	CAASSPIRGYTGSDKLIF	CATWDGFYYKKLFGSG

### Cells and cell lines

PBMCS were isolated by Ficoll-Paque (GE Healthcare, cat no. Cytvia 17-1440-03)

from buffy coats obtained from Sanquin Blood Bank). αβT cells were expanded from PBMCs using CD3/CD28 dynabeads (Thermo Fisher scientific, cat no. 40203D) and (1.7 × 10^3^ IU/ml of MACS GMP Recombinant Human interleukin (IL)-7 (Miltenyi Biotec, cat no. 130-095-361), and 1.5 × 10^2^ IU/ml MACS GMP Recombinant Human IL-15 (Milteny Biotec, cat no. 130-095-762). HL60, RPMI 8226, and SSC9 stably expressing GFP-luciferase was generated by a previously described retroviral transduction protocol (30). The plasmid containing the GFP and luciferase transgenes was kindly provided by Jeanette Leusen (UMC Utrecht, Utrecht, Netherlands). The following cell lines were obtained from ATCC between 2010 and 2018, HL60 (CCL-240), RPMI 8226 (CCL-155), SCC9 (CRL-1629) and Daudi (CCL-213). Freestyle 293-F cells (R790-07) were obtained from Invitrogen. ML-1, HL60, RPMI 8226 and Daudi were cultured in RPMI (Gibco, cat no. 12017599), 10% FCS (Bodinco), 1% Pen/Strep (Invitrogen, cat no. 11548876). Freestyle 293-F in Freestyle expression medium (Gibco, cat no. 10319322). SCC9 in DMEM (Gibco, cat no. 31966047) 10% FCS, 1% Pen/Strep.

### Expression and purification of bispecifics

Bap and His-tagged γδ_VAR_ –αCD3, γδ_VAR_, or γδ_ECTO_-αCD3 were expressed in 293 F cells. 293 F cells were cultured in Gibco Freestyle Expression medium, as transfection reagent Polyethylenimine (PEI) (25 kDa linear PEI, Polysciences, cat no. 23966-1) was used. Transfection was performed using 293 F cells at a density of 1.10^6 cells/ml mixed with 1.25 µg DNA, 3.75 µg PEI and per million cells. DNA and PEI were pre-mixed in freestyle medium (1/30 of transfection volume), incubated for 20 minutes, and added dropwise to the cell cultures. The cultures were maintained shaking at 37°C 5% CO_2._ To biotinylate the protein during expression, a vector containing the bacterial biotin ligase BirA was added to the transfection mix (10% of total DNA), and six hours after transfection, the medium was supplemented with 100 µM Biotin. Cell culture supernatant was harvested after 5 days and filtered through a 0.22 µm filter top (Milipore, Cat no. S2GPT02RE). Supernatant was adjusted to 25 mM Tris (Sigma Aldrich, cat no. 1185-53-1), 150 mM NaCl (Sigma Aldrich, 7647-14-5)

and 15 mM Imidazole (Merck, 288-32-4) (pH 8) and loaded on a 1 ml HisTrap HP column (GE healthcare, cat no. 17-5247-01) using the ÄKTA start purification system (GE healthcare). The column was washed with IMAC loading buffer (25 mM Tris,150 mM Nacl 15 mM Imidazole (pH 8), and protein was eluted using a linear imidazole gradient from 21 to 300 mM in 20 CV. Fractions containing the expressed protein were pooled, concentrated and buffer exchanged to TBS (25 mM tris, 150 mM NaCl, pH 8) using vivaspin 20 30kD spin columns (Sartorius, cat no. **VS2022**). Protein was diluted 100 times in IEX loading buffer (25 mM Tris pH 8), and loaded onto a HiTrap Q HP 1 ml column (GE healthcare, cat no. 17-1153-01) using the ÄKTA start purification system, for a second purification step. The column was washed with 10 column volumes IEX loading buffer, and protein was eluted using a linear NaCl gradient form 50 to 300 mM in 25 CV. Fractions containing the GAB were pooled, concentrated using vivaspin 20 30kD spin columns and examined by SDS-PAGE and staining with Instant blue protein stain (Sigma Aldrich, cat no. ISB1L). Protein concentration was measured by absorbance on Nanodrop and corrected for the Extinction coefficients. The protein was snap frozen and stored at -80°C and thawed before use.

### Beads coated with variable domains of the γ and δ chains (γδ_VAR_) for target cell staining

Biotinylated soluble γδ_VAR_ was mixed with 5-7µm streptavidin-coated UV-beads (Spherotech) in excess to ensure fully coated beads, 10 µg γδ_VAR_/mg microspheres. 7.5*104 cells, ML1 or K562, were incubated with 20 µl γδ_VAR_ -UV beads (0.33 mg beads/ml) for 30 minutes at RT. The mixtures were fixed by adding 20 µl 2% formaldehyde for 15 minutes. The samples were washed once with 1% formaldehyde and analyzed on a BD FACSCanto II (BD).

### Size exclusion chromatography and multi angle light scattering

Size exclusion chromatography was performed on a Yarra 3 uM SEC 3000 column (Phenomenex) using the high Performance Liquid Chromatography system (Shimadzu). The column was washed with SEC running buffer (100 mM Sodium Phosphate 150 mM NaCl pH 6.8) before loading of the samples. Protein samples were 5x diluted in SEC running buffer and filtered through a 0.22 uM centrifugal filter before loading on the column. For molecular weight characterization SEC was performed with online static light scattering (miniDAWN TREOS, Wyatt Technology) and differential refractive index (dRI, Shimadzu RID-10A) on a Shimadzu HPLC system. Data were analysed using the ASTRA software suite v.6.1.5 (Wyatt Technology).

### IFNγ ELISA/Elispot

15.000 (Elispot) or 50.000 (ELISA) effector cells and 50.000 target cells were incubated together with or without GAB (different concentrations) for 16 hours at 37°C 5% CO_2_. 30 or 100 µM pamidronate (calbiochem, cat no. 109552-15-0) was added to the target cells. For ELISA the supernatant was harvested after 16 hours, and the level of IFNγ was determined using the IFN gamma Human Uncoated ELISA Kit (Invitrogen, cat no. 15541107). For the Elispot assay the co-culture was done in nitrocellulose-bottomed 96-well plates (Millipore, cat no. MSIPN4550) precoated with α-IFNγ antibody (Mabtech, 3420-3-1000, clone 1-D1K 1:200). After 16 hours, the plates were washed with PBS and incubated with mAb7-B6-1 (II; Mabtech, cat no. 3420-6-1000) followed by Streptavadin-HRP (Mabtech, cat no. 3310-9) IFNγ spots were visualized with TMB substrate (Mabtech, cat no. 3651-10) and analyzed using A.EL.VIS ELISPOT Scanner and analysis software (A.EL.VIS).

### Luciferase based cytotoxicity

5000 or 10000 target cells stably expressing luciferase were incubated with T cells at a 3:1 or 5:1 T cell to target cell ratio, with different γδ_ECTO_-αCD3 concentrations (as indicated) in the presence of 30 or 100 µM pamidronate (calbiochem, cat no. 109552-15-0). After 16 hours, beetle luciferin (Promega, E1602) was added to the wells (125 µg/ml) and bioluminescence was measured on SoftMax Pro plate reader. The signal in treatment wells was normalized to the signal measured for targets and T cells only, which was assumed to represent 100% living cells.

## Results

### Variable domains of the γ and δ chains (γδ_VAR_) are poorly expressed as a single chain fragment

**Figure 1:**
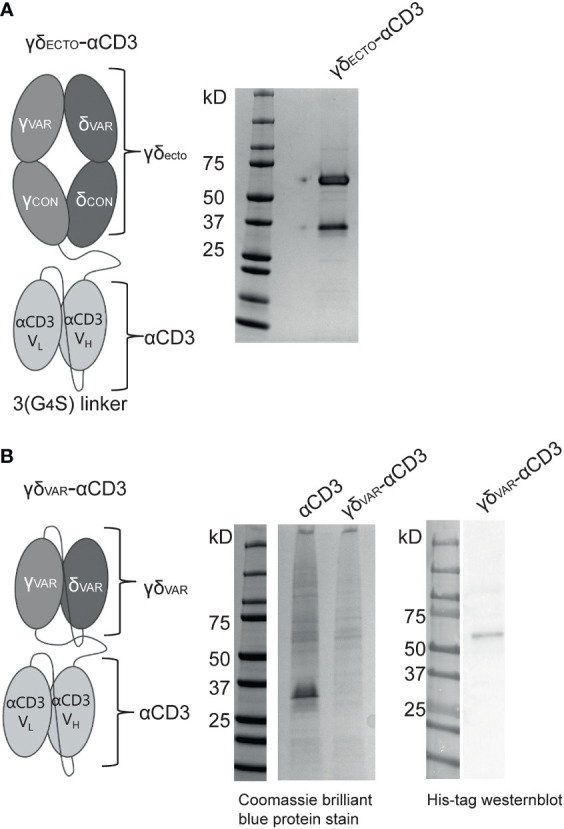
Expression of a γδ_Var_–αCD3 bispecific molecule **(A)** Schematic representation of the γδ_ecto_- αCD3, showing the extracellular (ecto) γδTCR (top), with the TCRγ chain connected to an anti-CD3 single chain variable fragment (αCD3) with the variable light (V_L_)and heavy (V_H_) and light chain (bottom) *via* a flexible linker. Purified GAB was run on SDS-page gel and stained with coomassie brilliant blue protein stain: visualizing the ectoγ-CD3scFV (59kD) and ectoδ chain (26 kD). **(B)** Schematic representation of the γδ_var_–αCD3 with the Vδ-Vγ single chain TCR fragment (scTv) (top) linked to an anti-CD3 scFv (bottom) *via* a flexible linker. After HIS-tag purification the CD3scFv and γδ_Var_ –αCD3 samples were run on SDS gel and visualized with coomassie brilliant blue protein stain. (left) or His-Tag western blot (right).

The GAB design published to date is a fusion of ectodomains of a γδ T cell receptor (TCR) to an anti-CD3 single chain variable fragment (γδ_ECTO_-αCD3) (**Figure 1A**) (13). We next explored strategies to increase the valency of GABs, in an effort to further increase potency. Multivalent tumor binding could be achieved, for example, by generating shorter single chain variable fragments as tumor- and T cell binding domains, and linking these in tandem with the desired stoichiometry (33). To test the feasibility of this approach, we constructed variable domains of the γ and δ chain (γδ_VAR_) linked to an anti-CD3 single chain variable (αCD3) fragment with 1:1 stoichiometry (γδ_VAR_-αCD3) (**Figure 1B**). γδ_VAR_-αCD3 and the αCD3 alone (as a positive control) were expressed in HEK293F cells, and protein production was evaluated on a SDS gel after His-tag purification. While there was a visible band for the αCD3 alone around 30kD, we did not observe expression of the γδ_VAR_-αCD3, which is expected at 62kD (**Figure 1B** left panel). We were able to visualize a band for the γδ_VAR_-αCD3 using Western blot, indicating that this design does result in expressed protein, but yields are not comparable to quantities produced for αCD3 alone (**Figure 1B** right panel).

### Stabilizing mutations reported from αβ variable T cell receptor single chains increase expression of γδ_VAR_-αCD3 by three-fold

For αβTCR-derived single chains, expression yields are often very low compared to antibody-derived single chains, due to aggregation and misfolding of the protein (33). Therefore, introduction of stabilizing mutations is, in general, required to achieve successful expression of αβTCR-derived single chains (34–36). These stabilizing mutations are often unique for each TCR, and are usually identified by large random mutagenesis PCR screens. In an attempt to identify a more broadly applicable engineering strategy, Richman et al. compared stabilizing mutations found for several different αβTCR-derived single chains, and identified amino acids that were mutated in more than one stabilized αβTCR-derived single chain (35). To determine which of the regular occurring stabilizing mutation in single chain αβTCR would be suitable to include in our γδ_VAR_, we aligned the sequences of variable domains of αβTCR 2C (PDB 1TCR) and γδTCR G115 (PDB 1HXM) (**Supplementary Figure 1A**) and their corresponding protein structures in PyMOL. Based on the location and chemical environment of the residues in the γδTCR and the potential benefit of mutations that are present in single chain αβTCRs, we selected six mutations to introduce in the γδTCR variable chains (γδ_VAR-MUT_) (**Supplementary Figure 1B**). Three out of five mutations in the gamma chain were localized in the region of the variable domain that interacts with the constant domain in the full length TCR. These three mutations have the potential to either change polarity/hydrophobicity (γK_13_V in orange and yI_99_S blue) or flexibility (γV_49_E in green) of the variable gamma chain (**Supplementary Figure 1B**) (35). Two other gamma chain mutations (γV_49_E in blue and γI_50_L in red) plus the delta chain mutation (δM_50_P in red) are located in in the variable γ- variable δ interface (in red, **Supplementary Figure 1B**).

**Figure 2:**
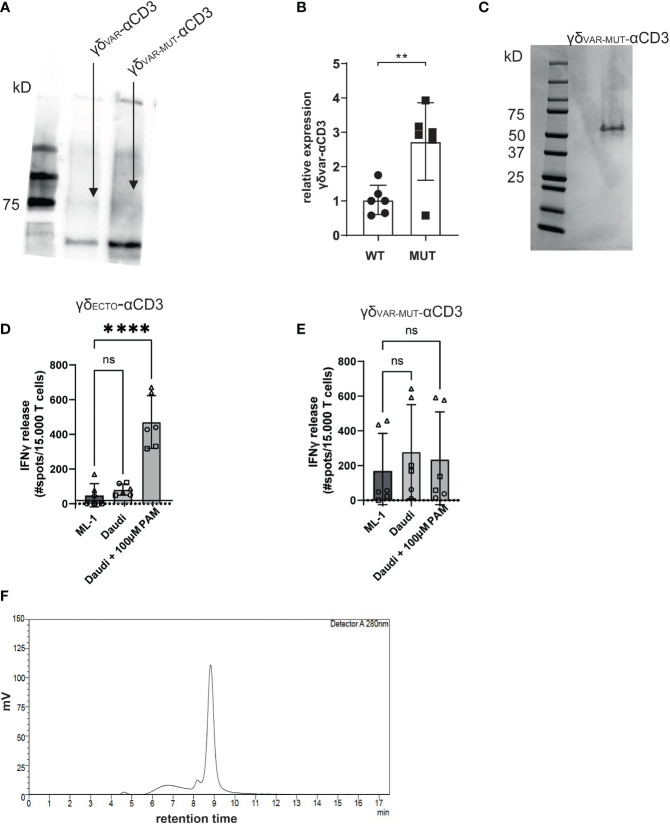
γδ_VAR-MUT_–αCD3 does not redirect T cells to tumor cells **(A)** γδ_Var_–αCD3 WT or with six stabilizing mutations, were expressed in HEK293F cells, and protein expression was visualized using His-Tag western blot. **(B)** Expression of γδ_Var_–αCD3 6mut relative to the WT γδ_Var_–αCD3. N=6 error bars represent SD, significance was calculated using an unpaired **p≤ 0.01. **(C)** SDS-PAGE analysis of purified γδ_VAR-MUT_–αCD3. D\E) T lymphocytes were co-incubated with **(D)** γδ_ECTO_-αCD3 or **(E)** γδ_VAR-MUT_–αCD3 (5-10 µg/ml) and target cells ML-1 or Daudi, -/+ 100 µM pamidronate (PAM). IFNγ release was measured by ELISPOT. The different symbols represent three different experiments (two technical replicates). N=3, error bars represent SEM, significance was calculated using one-way ANOVA, ns not significant p>0.05, ****p≤ 0.0001. **(F)** Size exclusion chromatogram of the γδ_VAR-MUT_–αCD3.

γδ_VAR_-αCD3 and γδ_VAR-MUT_-αCD3 were expressed in HEK293F cells, and protein production was evaluated by western blot (**Figure 2A**). Introduction of the six mutations approximately tripled the expression yield of γδ_VAR-MUT_-αCD3 when compared to γδ_VAR_-αCD3 (**Figure 2B**). Despite the rather modest increase in expression by only threefold, we next performed a large-scale production and purification of the γδ_VAR-MUT_-αCD3 (**Figure 2C**). To assess activity, the purified γδ_VAR-MUT_-αCD3 and γδ_ECTO_-αCD3 (as a positive control) were added to a co-culture of T lymphocytes, and the target cell line Daudi, previously shown to be recognized by γ9δ2 T cells (37). The unrecognized cell line ML-1 was used as a negative control, and additionally the Daudi cells were treated with the mevalonate pathway inhibitor pamidronate (PAM) to enhance γ9δ2TCR mediated recognition (30). While the γδ_ECTO_-αCD3 only induced T cell activation against Daudi cells treated with PAM (**Figure 2D**), the γδ_VAR-MUT_-αCD3 did not induce differential recognition of the target cell lines (**Figure 2E**). In one experiment the γδ_VAR-MUT_-αCD3 induced nonspecific T cell activation, which could imply the presence of larger protein aggregates that can trigger T cell activation without target cell engagement. Size exclusion chromatography (SEC) of γδ_VAR-MUT_-αCD3 protein confirmed that in addition to the monomeric γδ_VAR-MUT_-αCD3 peak at 9 minutes, a large proportion of the γδ_VAR-MUT_-αCD3, ~25%, was eluted before this monomeric peak, indicative of aggregated γδ_VAR-MUT_-αCD3 (**Figure 2F**).

**Figure 3:**
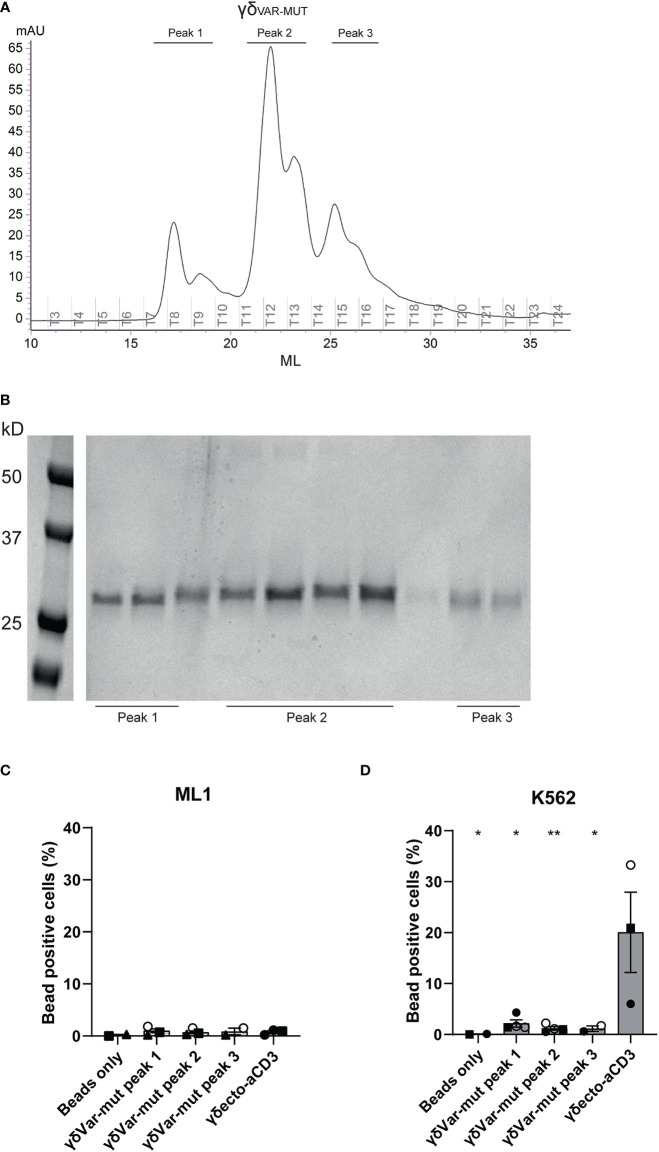
Expression and misfolding of single chain γδ_VAR-MUT_. **(A)** γδ_var_ with stabilizing mutations (γδ_VAR-MUT_) were expressed in HEK29F cells and purified using ion exchange chromatography **(B)** the different protein elution fractions after ion exchange chromatography (IEX) were run on SDS gel and visualized by coomassie brilliant blue staining C/D) Fluorescent beads were coated with the indicated IEX protein elution peaks of γδ_VAR-MUT_ or control γδ_ECTO_ and incubated with ML1 **(C)** and K562 **(D)** cells. Graph shows % beads positive cells. The different symbols represent different experiments. Closed symbols represent protein elution fractions from batch 1, open symbols represent protein elution fractions from batch 2. N=3, error bars represent SEM, significance was calculated using a multiple comparison one-way ANOVA, comparing all means to the mean of γδecto, *=p≤ 0.05 **= p≤ 0.01.

To assess the expression and folding properties of the γδ_VAR-MUT_ specifically, γδ_VAR-MUT_ was expressed in HEK293F cells and purified using ion exchange chromatography. The γδ_VAR-MUT_ was eluted in several peaks (**Figure 3A**), indicating that there is a variation in the physical properties of the protein, which could have an influence on its functionality. When the different fractions were evaluated on SDS gel, all contained the γδ_VAR-MUT_ (**Figure 3B**). We have previously shown that it is possible to assess γ9δ2 TCR binding to target cells by coating soluble γδ_ECTO_ on fluorescent streptavidin beads and evaluation of bead binding by flow cytometry (17). To test the γδ_VAR-MUT_ in the different elution peaks for binding activity, the protein fractions corresponding to the separate peaks were coated on fluorescent streptavidin beads and assessed for K562 target cell binding by flow cytometry, ML-1 cells were used as a negative control. No staining was observed for beads coated with any of the γδ_VAR-MUT_ elution peaks of the two cell lines, while beads coated with γδ_ECTO_ specifically stained K562 cells and not the negative control cell line ML-1 (**Figures 3C, D**). Based on these results we can conclude that, similar to previous findings for αβTCR-derived single chains, in order for a γδ_VAR_-αCD3 to be expressed and functional, extensive work would have to be performed to stabilize the γδ variable domain single chain format.

### γδ_ECTO_-αCD3 -dimer formation occurs naturally and is impacted by the linker length between the heavy and light chain of αCD3

**Figure 4:**
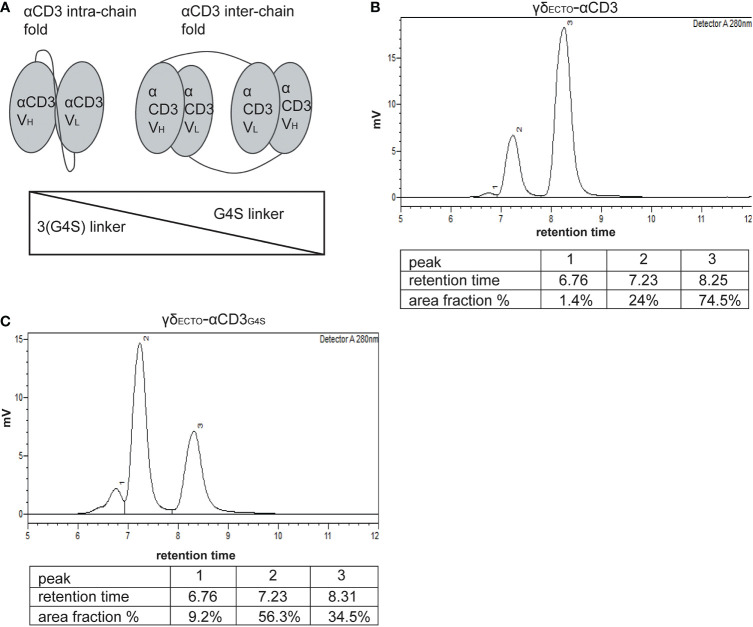
γδ_ecto_-αCD3-dimers are formed by αCD3 dimerization, which is influenced by linker length between the heavy and the light chain. **(A)** Schematic representation of αCD3 either folded by with intra-chain interaction (left) or with inter-chain interaction of two αCD3s (right). B+C) Size exclusion chromatography of γδ_ecto_-αCD3 comprising the linker 3(G4S) **(B)** or γδ_ecto_-αCD3_G4S_ **(C)**.

As alternative strategy to increase valency of GABs, we next considered possibilities to generate a multivalent GAB by using the original γδ_ECTO_-αCD3 design (**Figure 1A**). It has been reported previously that single chain fragments can cause protein oligomerization due to inter-chain variable heavy and light chain interactions, instead of the intended intra-chain heavy and light chain association (**Figure 4A**) (38, 39). To test whether the current γδ_ECTO_-αCD3 design harboring an anti-CD3 single chain variable fragment with the heavy and light chain linked with a 3(G4S) flexible linker (γδ_ECTO_-αCD3) results in multimerization of the γδ_ECTO_-αCD3 molecules, γδ_ECTO_-αCD3 were analyzed, using size exclusion chromatography (SEC) (**Figure 4B**). The SEC chromatogram of γδ_ECTO_-αCD3 showed three peaks, with the peak at the highest retention time (peak 3) containing the most protein, implying that there are indeed more size variants in the protein product. Separate analysis of the two major protein peaks (2 and 3) on SDS-PAGE showed the presence of both protein chains in the peaks, with no difference in relative signal intensity between the chains (**Supplementary Figure 2A**). The SEC was repeated with different protein batches, always resulting in a similar chromatogram, with a comparable ratio between the percentage area under the curve (AUC) of the 2 major peaks (**Supplementary Figure 2B**). Furthermore, varying the TCR sequence either by changing the CDR3 region of the Vδ2 or the complete Vγ9 or Vδ2 chain (Clone 5, 6_2, EPCR-reactive γδTCR) in the γδ_ecto_-αCD3, did not influence the ratio of percentage AUC of the two size variants (**Table 1** and **Supplementary Figure 2C**).

To determine the size of the GAB variants in both peaks we first used SEC-reference standards, containing 5 different molecules with known molecular weight. Based on the calibration curve the GAB variant peak 2 would have a molecular weight of around 310 kDa and the GAB variant in peak 3 would have a molecular mass of around 115 kDa (**Supplementary Figure 2D**). Assuming that the peak 3 would contain monomeric GAB, with a theoretical molecular mass of 85 kDa, this number deviates substantially. These large deviations in molecular mass are not uncommon when using SEC as the retention time is not only dictated by the size of the protein, but also by the shape (40). To formally determine the exact size of the γδ_ECTO_-αCD3 protein in the SEC peaks, we performed size exclusion chromatography with multi angle light scattering (SEC-MALS). The MALS analysis provided the molar masses for the 2 major sized peaks, with peak 2 consisting of a protein with a molar mass 176.7 kDa, and peak 3 of a protein with a molar mass of 88.45 kDa, corresponding to dimeric and monomeric γδ_ECTO_-αCD3 respectively (**Supplementary Figure 2E**), the small deviation from the theoretical molar mass, 171 kDa and 85.5 kDa, can be attributed to N-linked glycosylation of γδ_ECTO_-αCD3 (**Supplementary Figure 2F**). While not determined in the SEC-MALS analysis, due to the small size, this means that peak 1 most likely contains trimerized γδ_ECTO_-αCD3.

One of the factors influencing the single chain folding is the length of the linker between the two variable chains, with shorter linkers sterically hindering intra-chain interaction and thereby promoting inter-chain interactions (**Figure 4A**). Therefore, the flexible linker between the heavy and light chain of αCD3 was shortened from 15 to 5 amino acids (3(G4S) to G4S, γδ_ECTO_-αCD3_G4S_). After production and purification, a sample of the γδ_ECTO_-αCD3_G4S_ was analyzed by SEC (**Figure 4C**), showing an increase in the relative amount of dimeric γδ_ECTO_-αCD3_G4S_ to over 50% of the total protein.

We conclude that it is possible to enhance the formation of naturally dimerized γδ_ECTO_-αCD3 from approximately 20%, to over 50% by decreasing the linker length. Of note, there was no clear indication that larger aggregated oligomers, which could potentially cause non-specific T cell activation as seen for the γδ_VAR_-αCD3, are present in either γδ_ECTO_-αCD3 product.

### γδ_ECTO_-αCD3_G4S_ production is less efficient than γδ_ECTO_-αCD3

**Figure 5:**
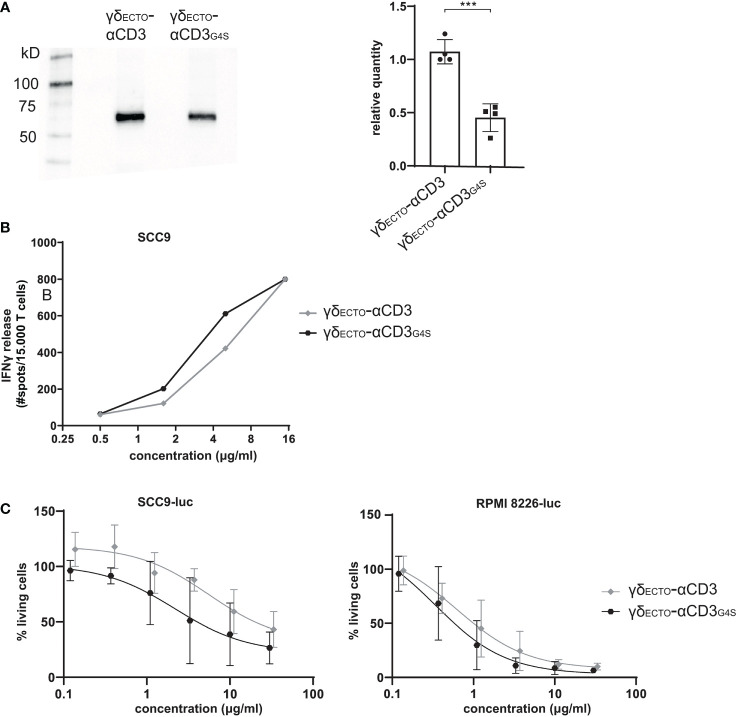
Functionality and expression of γδ_ecto_-αCD3 and γδ_ecto_-αCD3_G4S_. **(A)** Westernblot of unpurified expression medium with γδ_ecto_-αCD3 and γδ_ecto_-αCD3_G4S_ GAB. The ecto γ_ecto-_αCD3 chain is visualized by α-HIS western blot B) T lymphocytes were co-incubated with SCC9 target cells in the presence of PAM (100 µM) and γδ_ecto_-αCD3_3(G4S)/G4S_ (0.5-15 µg/ml) overnight. IFNy was measured by ELISPOT C) Effector and luciferase transduced RPMI 8226 were co-incubated for 16 hours in the presence and absence of γδ_ecto_-αCD3_3(G4S)/G4S_ at different concentrations and PAM (30 μM). Percentage viable cells was determined by comparing luminescence signal to the no γδ_ecto_-αCD3 condition, representing 100% viability. N=4 **(A)**, N=2 **(B)**, N=4 **(C)**, error bars represent SD. Significance was calculated using an unpaired T-test ***P≤ 0.001.

Unfortunately, although the shorter G4S linker led to a higher percentage of dimer formed during protein expression, it also decreased total protein expression, as shown in a side by side comparison of expression medium of γδ_ECTO_-αCD3 and γδ_ECTO_-αCD3_G4S_ by western blot (**Figure 5A**). On average, the relative expression of the γδ_ECTO_-αCD3_G4S_ compared to γδ_ECTO_-αCD3 was decreased by two-fold, meaning that overall, while the G4S linker approximately doubles the proportion of formed dimer, it also causes a two-fold decrease in protein expression.

### γδ_ECTO_-αCD3-dimers are functionally superior to monomers

Despite the lower efficiency in the production of γδ_ECTO_-αCD3_G4S_ compared to γδ_ECTO_-αCD3, we tested whether, without further purification of the monomer and dimer fraction, differences in the activity between both constructs could be observed. γδ_ECTO_-αCD3 and γδ_ECTO_-αCD3_G4S_ were therefore titrated in a co-culture of T lymphocytes and SCC9 target cell line, and IFNγ release was determined by ELISPOT (**Figure 5B**). The γδ_ECTO_-αCD3_G4S_ showed a slight increase in functional avidity, defined as IFNγ release, compared to the γδ_ECTO_-αCD3, probably due to the higher percentage of dimer present in the γδ_ECTO_–αCD3_G4S_ protein product. Next, we also tested the γδ_ECTO_-αCD3 and γδ_ECTO_-αCD3_G4S_ for direct target cell killing, using a luciferase-based cytotoxicity assay. Luciferase transduced target cell lines (RPMI8226 and SCC9) were co-cultured with T cells and different concentrations of γδ_ECTO_-αCD3 and γδ_ECTO_-αCD3_G4S,_ and the amount of viable cells was determined (**Figure 5C**). Again, we observed a slight, but not significant, increase target cell killing of the γδ_ECTO_-αCD3_G4S_ compared to γδ_ECTO_-αCD3.

We hypothesized that the lack of significance in activity was most likely a consequence of the still rather limited difference in the amount of dimers (20% and 50% dimer; **Figures 4B, C**), which made it difficult to formally asses the true value of dimers, when compared to monomers. As the shortening of the G4S linker also significantly decreased the expression efficiency of the γδ_ECTO_-αCD3 protein, we decided to assess the impact of purified dimer and monomer fractions derived from the original design, namely γδ_ECTO_-αCD3.

**Figure 6:**
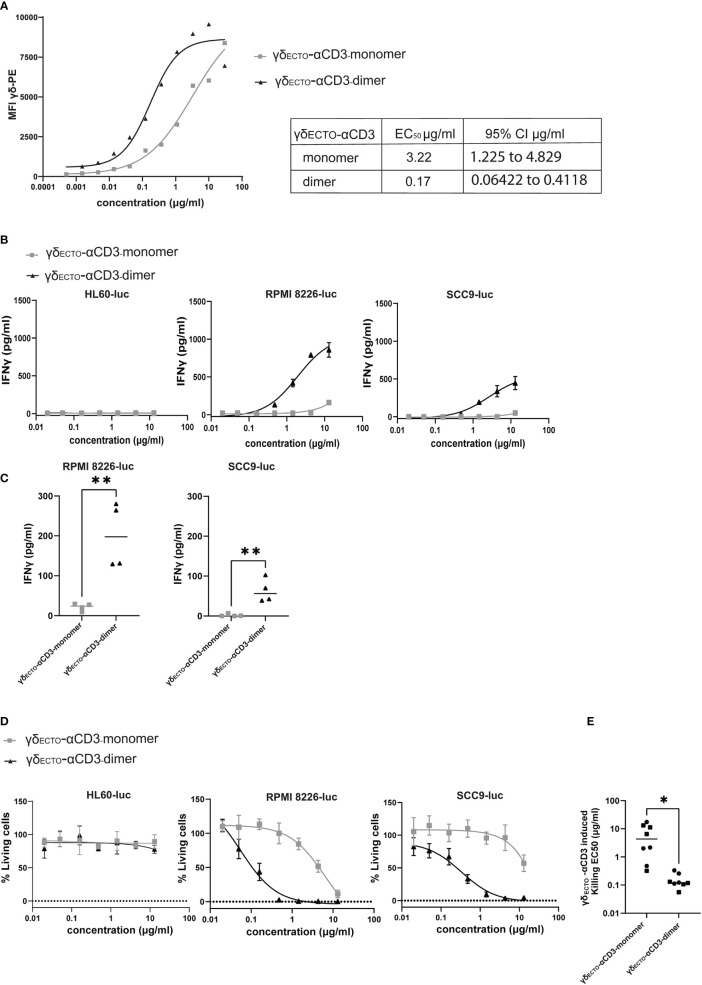
γδ_ecto_-αCD3-dimers are functionally superior **(A)** Coating of T lymphocytes with γδ_ecto_-αCD3-monomers or dimers, followed by staining with fluorochrome labeled anti pan-γδ antibody. MFI was measured by flow cytometry, representative figure is shown N=3. **(B)** T cells were incubated with target cells, PAM (30 μM) and γδ_ecto_-αCD3-monomers or dimers (0.02-15 µg/ml) for 20 hours. IFNγ release was measured by ELISA. Plots present mean + SD of duplicates of a representative assay, N=4 for all cell lines. **(C)** IFNγ release at a γδecto-αCD3 concentration at 0.6 µg/ml (as in B) for RPMI8226-luc and SCC9-luc. Unpaired t test was used to determine significance between the γδecto-αCD3 monomer and dimer conditions, ** P-value <0.01 (GraphPad Prism). Each dot represents the mean of biological replicate. **(D)** T lymphocytes and luciferase transduced HL60, RPMI8226, and SCC9 target cells were co-incubated for 20 hours in the presence and absence of γδ_ecto_-αCD3-monomers or dimers at different concentrations and PAM (10 μM) at an E:T ratio of 5:1. Percentage viable cells was determined by comparing luminescence signal to the no GAB condition, representing 100% viability. Plots present mean + SD of triplicates of a representative assay, N=4 for all cell lines. **(E)** EC50 for each killing assay was determined in GraphPad Prism for RPMI8226-luc and SCC9-luc. Unpaired t test was used to determine significance between the γδecto-αCD3 monomer and dimer conditions, * P-value <0.05 (GraphPad Prism).

Preparative size exclusion chromatography was used to separate monomeric and dimeric γδ_ECTO_-αCD3. As dimeric γδ_ECTO_-αCD3 are, in theory, not only bivalent for tumor binding, but also for CD3 binding, the binding properties of monomeric and dimeric γδ_ECTO_-αCD3 to T lymphocytes were first evaluated. Purified monomeric and dimeric γδ_ECTO_-αCD3 were titrated and incubated with T lymphocytes, followed by a secondary staining using fluorochrome labeled panγδ-TCR antibody (**Figure 6A**). A comparison of the MFI between the dimer and the monomer showed an increase in T cell binding at lower γδ_ECTO_-αCD3 concentrations for the dimeric form, compared to the monomer. This could be attributed to an increase in the CD3 binding avidity of the dimer protein, but might also be partially explained by the presence of two binding epitopes for the panγδ-TCR antibody in each dimeric γδ_ECTO_-αCD3.

To test whether dimeric GABs are more potent than monomeric GABs to specifically activate T lymphocytes, we titrated monomeric or dimeric γδ_ECTO_-αCD3 in a co-culture with T cells and target cells, either the non-recognized cell line HL60 (37) or one of the previously used recognized cell line RPMI8226 or SCC9. This titration showed that the dimeric γδ_ECTO_-αCD3 was more potent compared to monomeric γδ_ECTO_-αCD3, inducing more IFNγ release compared to monomer in a co-culture with recognized target cells, RPMI8226 and SCC9, while no IFNγ release was detected in the presence of the non-recognized target cell line HL60 for either dimeric or monomeric γδ_ECTO_-αCD3 (**Figure 6B**). IFNγ release by T cells was significantly increased for dimeric γδ_ECTO_-αCD3 at concentrations ≥ 0.6 µg/ml when co-cultured with RPMI8226 and SCC9 (**Figure 6C**).

A luciferase based killing assay was performed to directly compare the dimers and monomers of γδ_ECTO_-αCD3 for the ability to induce target cell lysis. Luciferase transduced HL60, RPMI8226, and SCC9 targets cells were co-cultured with T cells and an increasing protein concentration. Neither monomeric nor dimeric γδ_ECTO_-αCD3 did induce T cell mediated killing of the non-recognized target cell line HL60, in line with the lack of T cell activation in the cytokine release assay. Dimeric γδ_ECTO_-αCD3 induced more target cell killing at lower protein concentrations for both tested recognized target cell lines RPMI8226 and SCC9, while monomeric γδ_ECTO_-αCD3 induced efficient target cell lysis only at higher concentrations (**Figure 6D**), which is also reflected in the significant difference in EC_50_ between γδ_ECTO_-αCD3 monomer and dimer (**Figure 6E**). In conclusion, our data shows that increasing the avidity of the γδTCR binding in the GAB format enhanced the potency *in vitro*, with the dimeric form of γδ_ECTO_-αCD3 being superior to the monomeric form. Furthermore, bivalent CD3 engagement alone does not cause T cell activation, but requires target cell engagement.

## Discussion

In this report we have explored different possibilities to increase the binding valency of previously described GABs (13). We show that dimers are a natural by-product of the recently reported γδ_ECTO_-αCD3 design, and that γδ_ECTO_-αCD3-dimers have higher activity when compared to γδ_ECTO_-αCD3-monomers. However, all efforts to generate meaningful amounts of γδ_ECTO_-αCD3-dimers, and strategies to increase valency by generating single chain formats derived from the variable domains the of the γδTCR (γδ_VAR_-αCD3) were jeopardized by the lack of efficiency, and misfolding during protein production.

Identifying a means to increase valency of the GABs without compromising protein yields will be critical for further clinical translation, in order to guarantee sufficient amounts of protein during GMP-grade production, and to enter a clinical trial with the most active compound. There are several other TCEs described in literature that are multivalent in tumor binding, for example tandem diabodies (41) with two separate chains interacting to form four linked single chain variable fragments, or immunoglobulins with one or two extra antigen binding fragments attached (42, 43). These designs are, however, not easily translated to the GAB format, as we have shown here that the expression yield of a single chain γδ_VAR_ was very low, and most of the expressed single chain γδ_VAR_ was misfolded and not functional. This is not surprising, given the long journey required to develop stabilized αβTCR-derived single chains (34–36). While we have shown that the introduction of mutations, based on stabilizing mutations for αβTCR-derived single chains, increased expression efficiency of γδ_VAR_ three-fold, further attempts to stabilize the single chain γδ_VAR_ will be needed. Due to the inherent differences in sequence between variable domains of the αβ and γδ chains, non-optimal choices might have been made.

We next focused on the original γδ_ECTO_-αCD3 design because of its sufficient stability, and observed spontaneous formation of monomers and dimers during expression. γδ_ECTO_-αCD3-dimers are most likely formed by dimerization of αCD3 domains from two γδ_ECTO_-αCD3 molecules. This assumption was supported by our observation that dimer formation could be enhanced by shortening the linker length between the variable heavy and light chain of the αCD3 fragment (γδ_ECTO_-αCD3_G4S_). With a linker of 15 amino acids 20% of the γδ_ECTO_-αCD3 protein was dimerized, which could be increased to over 50% by decreasing the linker length to only 5 amino acids in γδ_ECTO_-αCD3_G4S_. The functional benefit of increased dimerization of the γδ_ECTO_-αCD3_G4S_ was rather limited, and significant functional benefits could only be observed for γδ_ECTO_αCD3-dimers when comparing purified dimers with purified monomers. Introduction of the shorter linker also decreased expression efficiency of the γδ_ECTO_-αCD3_G4S_, which could be because this shorter linker is also more prone to cause larger misfolded oligomers that will be excluded during protein purification (38). Further clinical testing and development of the multivalent GABs using this αCD3 dimerized format is therefore not feasible. Addition of a dimerization domain to the C terminus of the αCD3 to induce association of two monovalent γδ_ECTO_-αCD3 to form a dimer, as reported for other TCEs, could be a more efficient alternative (27, 44, 45).

Common dimerization domains cause symmetric dimerization of two identical molecules, thereby inducing a symmetric multivalent γδ_ECTO_-αCD3 containing two tumor engaging- and two CD3 binding domains. We have shown in this report that the dimerized αCD3 of γδ_ECTO_-αCD3 did not result in a non-specific T cell activation, in line with observations for other TCE harboring two CD3 binding domains (41, 45). However, dual CD3 engagement and the risk for subsequent target cell independent T cell activation remains a concern in the field, and needs to be thoroughly investigated when designing a next generation of TCEs (29). In this light, the dock-and-lock method would be an interesting strategy to explore for the creation of a 2:1 valency GAB (44).

Despite the fact that our data imply that dimers are the preferred choice for further exploration to improve the potency of GABs, a potential downside of the introduction of additional multimerization domains in the GAB is that these larger multimers might substantially increase the space between the tumor- and CD3-binding domains, which could lead to a decreased activation efficacy, due to suboptimal immune synapse distances. The remarkable high potency of the FDA approved TCE blinatumomab is partially attributed to its small size, causing the formation of very tight immune synapses that are indistinguishable from naturally formed TCR-MHC synapses after target and T cell engagement (46). The overall effect of TCE size on efficacy is, however, also dependent on the exact binding epitope on the ligand. Chen et al. showed that while a smaller TCE was more efficient when binding to a membrane distal epitope, this effect was reversed when the binding epitope was more membrane proximal (47). As the exact binding mechanism and ligands for the γ9δ2 TCR are not yet completely elucidated (17), the optimal size and design for GABs is hard to predict, and is probably best determined by an experimental approach.

In conclusion, our data imply that dimerization of GAB is an interesting strategy for further preclinical development, however the road towards clinical translation is challenging, as engineering meaningful yields of dimers remains challenging.

## Data availability statement

The raw data supporting the conclusions of this article will be made available by the authors, without undue reservation.

## Author contributions

EvD, DXB and JK wrote the paper. EvD, MN, LK, JZ, PHL and DXB performed experiments. All authors contributed to the article and approved the submitted version.
