# Supplementary material for: The making of multivalent gamma delta TCR anti-CD3 bispecific T cell engagers
Source: Front Immunol. 2023 Jan 5;13:1052090. doi: 10.3389/fimmu.2022.1052090 (PMC9851377; doi:10.3389/fimmu.2022.1052090)
Supplement: Supplementary file 1 [file DataSheet_1.pdf]

Supplementary Figures for:

The making of multivalent Gamma Delta TCR Anti-CD3 bispecific T cell engagers

Eline van Diest[1#], Mara J.T. Nicolasen[1#], Lovro Kramer[1], Jiali Zheng[1], Patricia Hernández-López[1], , Dennis X Beringer[1*], Jürgen Kuball[1,2*].

1. Center for Translational Immunology, University Medical Center Utrecht, Utrecht University, The Netherlands
2. Department of Hematology, University Medical Center Utrecht, Utrecht University, The Netherlands
[#]These authors share first authorship
*These authors share senior authorship

Corresponding author:
Jürgen Kuball
Email: j.h.e.kuball@umcutrecht.nl

Content:

- Supplementary Figure 1
- Supplementary Figure 2

Supplementary Figure 1

A

Variable Alpha/Delta

α2C .QSVTQPDARVTVSEGASLQLRCKYSYSA......TPYLFWYVQYPRQG**L**QLLLKYYSG..DPVVQGV.....

δG115 AIELVPEHQTVPVSIGVPATLRCSMKGEAI....GNYYINWYRKTQGNT**M**TFIYREK.......DIYGPGFK.

α2C NGFEAEFSKSNSSFHLRKASVHWSDSAVYFCAVSGF......ASALTFGSGTKVIVLP..

δG115 DNFQGDIDIAKNLAVLKILAPSERDEGSYYCACDTLGMGGEYTDKLIFGKGTRVTVEP..

Variable Bèta/Gamma

β2C EAAVTQSPRNKV**A**VT**G**GKVTLSCNQTNNH.......NNMYWYRQDTGH**G**LRLIHYSYG....

γG115 AGHLEQPQISST**K**TL**S**KTARLECVVSGITI....SATSVYWYRERPGE**VI**QFLVSISYD...

β2c AGSTEKGDIP.DGYKASRP.SQENFSLILELATPSQ**T**SVYFCASGGG......GTLYFGAGTRLSVL

γG115 GTVRKESGIPSGKFEVDRIPETSTSTLTIHNVEKQD**I**ATYYCALWEAQQELGKKIKVFGPGTKLIIT

B

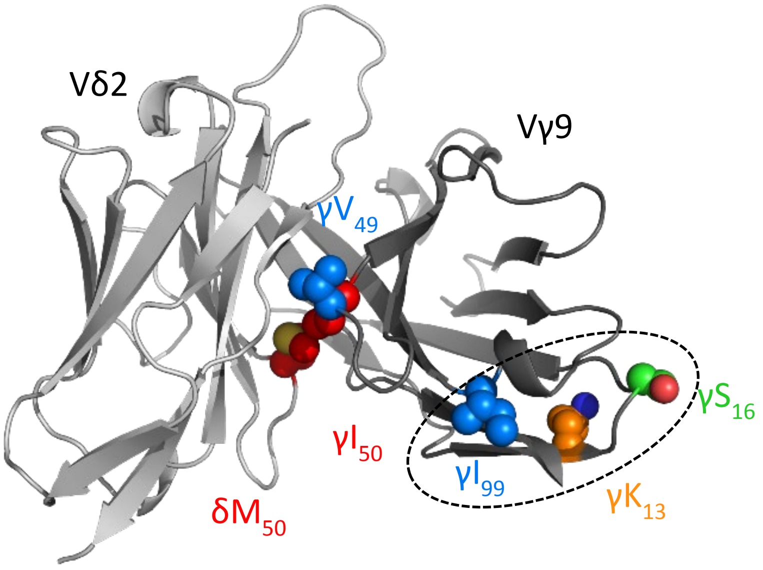

Supplementary Figure 1. Alignment of variable αβ- and γδ-chains and structure of G115 TCR with selected residues to be mutated to generate $γδ_{var-mut}$**.** A) Example alignment of a variable αβTCR 2C, (PDB 1TCR) and γδTCR G115 (PDB 1hxm). The selected frequently mutated amino acids identified in αβ variable T cell receptor single chains by Richman et al. are indicated in bold. Corresponding amino acids in the γ9- or δ2-chains of the G115 TCR are indicated in bold and color coded as shown in B. B) Structure of G115 TCR with selected AA highlighted in spheres, the dashed oval indicates the would-be Vγ-Cγ interface. (I) $γI_{99}S$; in blue: Changing the hydrophobic isoleucine to the more polar serine could make the variable gamma chain more stable when solvent exposed, a serine at this position is also highly conserved in Vβ genes of human and mouse. (II) $γK_{13}V$; in orange: Changing the positively charged amino-acid lysine to the smaller and more hydrophobic valine could potentially stabilize the variable gamma domain, because the valine can point more inwards and can be buried within the variable domain. Moreover, in many stabilized TCRs and in all variable heavy and light genes there is a small hydrophobic residue at this position (III) $γS_{16}G$; in green: most antibodies have at glycine at this position, which could be important for flexibility. (IIII) $γV_{49}E$; in blue: glutamic acid is the second most common amino acid at this position in the Vβ gene, and in two single chain αβTCRs a mutation from glycine to glutamic acid resulted in more stable protein. (V+VI) $γI_{50}L$+ $δM_{50}P$; in red: The introduced leucine and proline in the gamma and delta chain respectively could interact and potentially stabilize the variable gamma- delta interface.

Supplementary Figure 2

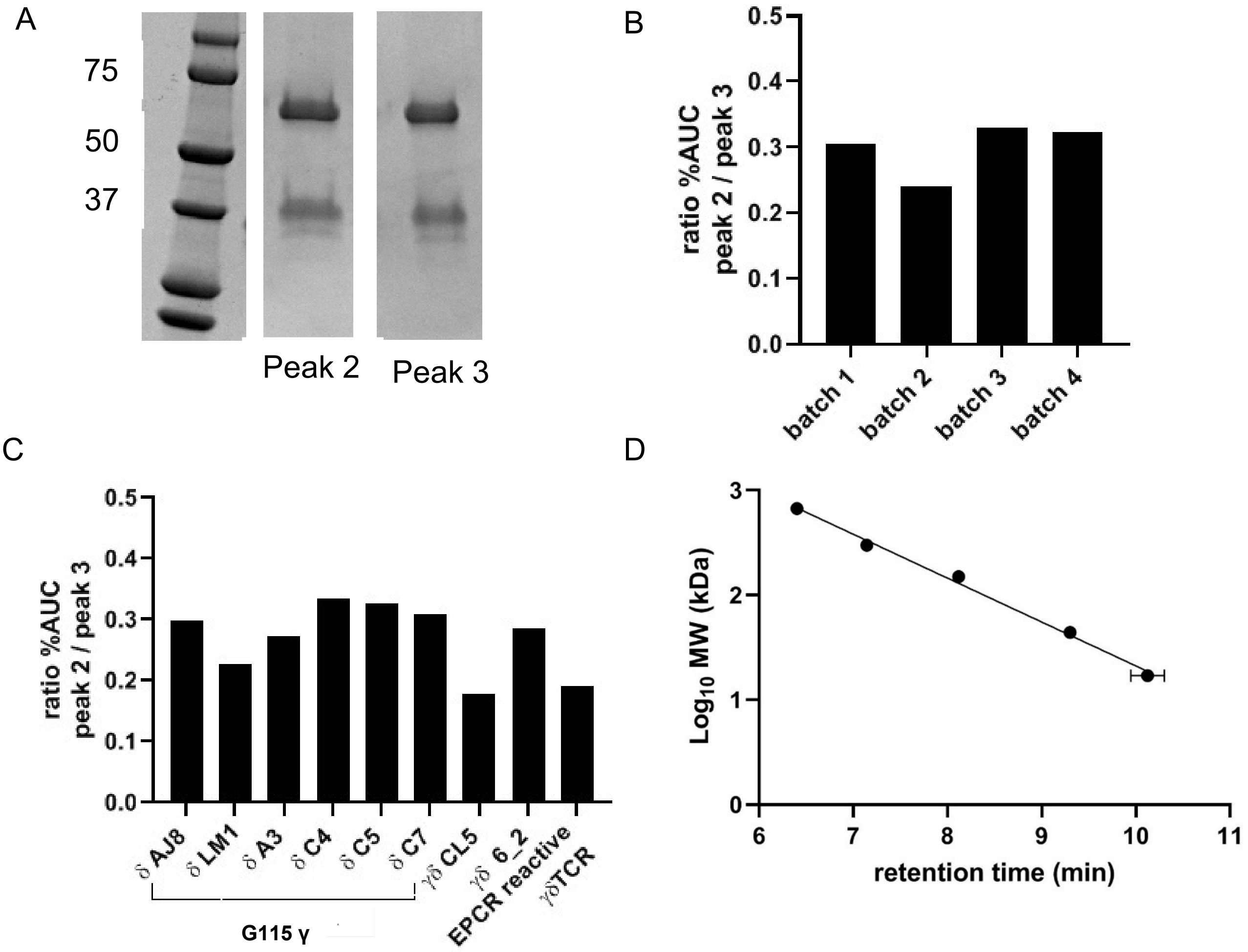

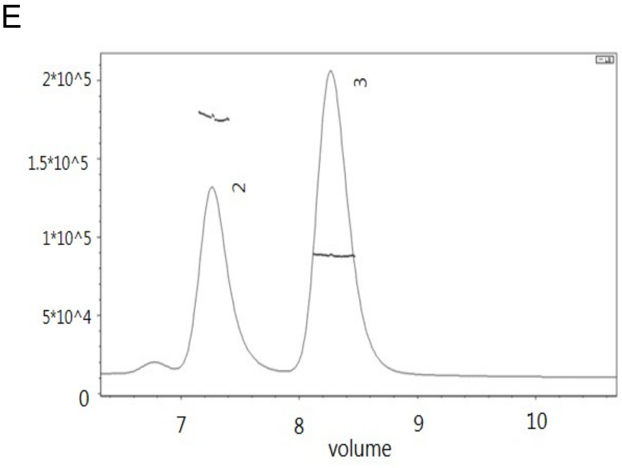

Peak	2	3
Molar mass (g/mol)	1.767×10^5	8.845×10^4
Confidence interval	± 0.177%	± 0.467%
	Dimer	Monomer

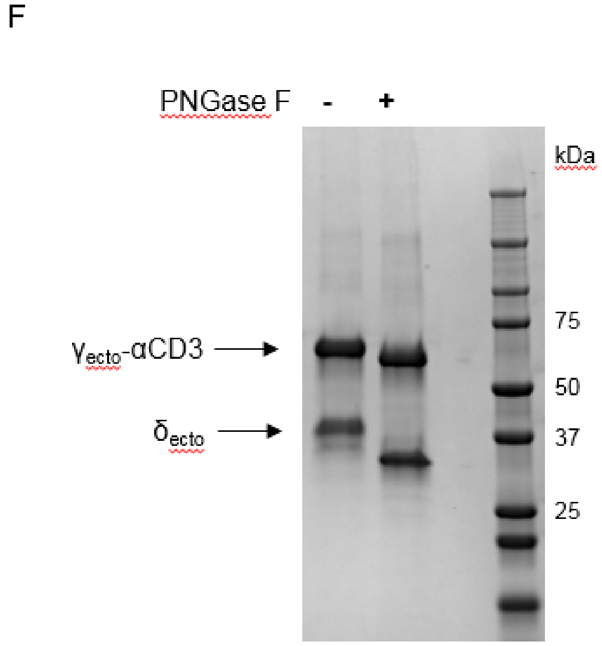

Supplementary Figure 2. γδ$_{ecto}$-αCD3-dimer formation is reproducible and independent from γδTCR sequence A) γδ$_{ecto}$-αCD3 purified protein from SEC peak 2 and 3 was run on SDS-page gel under reducing conditions and stained with coomassie brilliant blue protein stain B+C) The ratio between the % area under the curve (AUC) of the SEC peak 2 and 3 is plotted after size exclusion chromatography of B) different batches of γδ$_{ecto}$-αCD3 C) γδ$_{ecto}$-αCD3 derived from different γδ TCRs (AJ8 to C7 GAB: unique δ2 chain+ γ9 chain from G115 TCR, 6_2+CL5 GAB: unique γ9δ2 TCRs and C132 GAB: unique γ4δ5 TCR) D) Retention times of SEC mass standards (AL0-3042, Phenomenex) plotted against their mass (in kDa) each point represent the mean of 4 separate SEC runs, error bar represents the standard deviation. Linear regression was done in GraphPad Prism v9.30; R^2= 0.9948; "$Log_{10}MW$= -0.4190*(ret.time) + 5.514" E) SEC-MALS experiment to determine molecular size of protein in peak 2 and 3 visible after SEC, determining that the peak 2 corresponds to γδ$_{ecto}$-αCD3-dimer (1.77 $\times 10^5$ g/mol) while peak 3 consists of γδ$_{ecto}$-αCD3-monomer form (8.85$\times 10^4$ g/mol). F) SDS-PAGE analysis of the deglycosylation with PNGaseF under reducing conditions of γδecto-αCD3.
